# The lncRNA SEMA3B-AS1/HMGB1/FBXW7 Axis Mediates the Peritoneal Metastasis of Gastric Cancer by Regulating BGN Protein Ubiquitination

**DOI:** 10.1155/2022/5055684

**Published:** 2022-02-27

**Authors:** Guoquan Huang, Zhenxian Xiang, Haitao Wu, Qiuming He, Rongzhang Dou, Chaogang Yang, Jialin Song, Sihao Huang, Shuyi Wang, Bin Xiong

**Affiliations:** ^1^Department of Gastrointestinal Surgery & Department of Gastric and Colorectal Surgical Oncology, Zhongnan Hospital of Wuhan University, Wuhan, China; ^2^Department of Gastrointestinal Surgery, Central Hospital of Enshi Tujia and Miao Autonomous Prefecture, Enshi, China; ^3^Hubei Cancer Clinical Study Center, Wuhan, China

## Abstract

Peritoneal metastasis (PM) is one of the main causes of a poor prognosis in patients with advanced gastric cancer (GC). lncRNAs have been confirmed to play a very crucial role in the occurrence, development, and metastasis of many human cancers, including gastric cancer. However, the mechanism of lncRNA in PM of GC is rarely studied. We explored the mechanism of PM of GC through lncRNA gene sequencing and protein profiling analysis to detect PM-associated lncRNAs and proteins. A quantitative reverse transcription polymerase chain reaction (qRT-PCR) was performed to identify the mRNA expression of SEMA3B-AS1 and BGN in GC tissues and adjacent normal tissues. The biological function of SEMA3B-AS1 in the PM of GC was identified through gain- and loss-of-function assays. Chromatin isolation by RNA purification (ChIRP), RNA immunoprecipitation (RIP), RNA pull-down, luciferase reporter, and coimmunoprecipitation (co-IP) assays was carried out to demonstrate the potential mechanism between SEMA3B-AS1 and its downstream genes, including HMGB1, FBXW7, and BGN. Finally, the biological function of SEMA3B-AS1 was demonstrated in animal experiments. The mRNA expression level of SEMA3B-AS1 was downregulated in GC and PM tissues compared to normal stomach tissues; however, BGN was highly expressed at the mRNA level. SEMA3B-AS1 was closely related to PM and the overall survival (OS) of GC patients. Functionally, the overexpression of SEMA3B-AS1 was related to GC progression, PM, and prognosis. Mechanistically, SEMA3B-AS1 could combine with HMGB1 to regulate the transcription of FBXW7, thus facilitating the ubiquitination of BGN. In conclusion, our study demonstrated that the SEMA3B-AS1/HMGB1/FBXW7 axis plays an inhibitory role in the PM of GC by regulating BGN protein ubiquitination. It also provides a new biological marker for the diagnosis and treatment of the PM of GC.

## 1. Introduction

Gastric cancer (GC) is one of the leading causes of cancer-related death worldwide [1, 2]. Although great progress has been made in the treatment of GC, the prognosis of the peritoneal metastasis (PM) of GC remains poor [3, 4]. Recent studies have demonstrated that PM accounts for approximately 53%-66% of cases of GC distant metastasis [5–7]. At present, the mechanism of the PM of GC remains unclear, and there is a lack of effective means for the early diagnosis and treatment of PM. Therefore, it is urgent for us to further explore the molecular biological mechanism of the PM of GC and find new diagnostic and therapeutic markers.

Long noncoding RNAs (lncRNAs) play a vital role in the occurrence, development, invasion, and metastasis of cancer. lncRNAs are more than 200 nt in length and have a conserved secondary structure, and most of them do not have protein-coding function [8]. Existing evidence demonstrates that lncRNAs have crucial biological functions. They can interact with proteins, DNA, and RNA and participate in the regulation of a variety of biological processes [9, 10]. They can also regulate gene expression through various mechanisms, including epigenetic regulation, chromatin modification, transcription, posttranscriptional regulation, and protein metabolism [11–13]. Huang et al. found that lncRNAs can interact with cancer stem cells and then induce cancer metastasis and recurrence [14]. At present, the role of lncRNA in PM of GC remains to be further explored. According to the lncRNA sequencing in primary GC tissues and GC PM tissues, SEMA3B-AS1 was one of the most downregulated lncRNAs in PM tissues. Mass spectrometry (MS) analysis of SEMA3B-AS1 pull-down further revealed that mobility group box 1 (HMGB1) was an interacting protein of SEMA3B-AS1. Evidence demonstrated that HMGB1 is a multifunctional molecule in cell homeostasis. In the nucleus, HMGB1 plays an important protective role in the process of DNA replication, transcription, and chromatin remodeling, thus regulating DNA damage repair and maintaining genome stability as a DNA chaperone [15]. However, HMGB1 could promote the release of cytokines such as IL-6 and IL-8 by activating MAPK- and MyD88-dependent NF-KB pathways in the extracellular matrix, thus stimulating tumor cell proliferation, angiogenesis, epithelial-mesenchymal transition (EMT), invasion, and metastasis [16]. Tumor suppressor genes such as F-box and WD repeat domain-containing 7 (FBXW7), which we have previously studied, are usually inactivated after the occurrence of GC, and the body will issue instructions for gene repair, which allows tumor suppressor-related lncRNAs to recruit and bind genes or proteins that can repair DNA damage [15, 17–19]. Bioinformatics analysis revealed that HMGB1 was one of the transcription factors of FBXW7. Thus, whether SEMA3B-AS1 could combine with HMGB1 to regulate the transcription of FBXW7 remains unknown.

As is known to all, FBXW7 could regulate the ubiquitination of downstream protein [20]. Then, we find that the BGN protein is the ubiquitination substrate of FBXW7 through UbiBrowser website prediction. The BGN protein, which is a key member of the leucine-rich proteoglycan family and is mainly located in the cellular stroma, is localized on the long arm of human X chromosome Xq13-qter [21]. It is linked to a variety of human cancers and associated with the aggressive growth and metastasis of tumors, such as GC and colon cancer [22–24]. To our surprise, MS and bioinformatics analysis found that BGN is upregulated in PM of GC tissues, and its high expression correlates with metastasis and poor prognosis of GC patients. Therefore, whether FBXW7 could regulate the ubiquitination of BGN deserve to be further elucidation.

In current research, we found that SEMA3B-AS1 was downregulated in PM of GC, and its lower expression correlates with PM of GC and poor prognosis of GC patients. Function analysis revealed that SEMA3B-AS1 inhibited proliferation, migration, and invasion of GC. Mechanistically, SEMA3B-AS1 could combine with HMGB1 to regulate the transcription of FBXW7, thus facilitating the ubiquitination of BGN. Our study provides a new biological marker for the clinical diagnosis and treatment of patients with PM.

## 2. Methods and Materials

### 2.1. Tissue Samples and Patients

Fifty pairs of GC tissues and paracancerous normal tissues were obtained from the specimen bank of the Cancer Institute of Zhongnan Hospital of Wuhan University. All patients were diagnosed with GC according to the American Joint Committee on Cancer (AJCC) tumor staging manual and histopathological assessment [25], and none of them received preoperative radiotherapy or chemotherapy. All patients provided informed consent for the procedures, which were approved by the Internal Review and Ethics Committee of Zhongnan Hospital of Wuhan University (Wuhan, China; Ethical Approval Number: 2019079).

### 2.2. Immunohistochemistry (IHC) and Immunofluorescence (IF)

IHC: the specimens were fixed in 10% formaldehyde within 30 minutes and then paraffin embedded. Before the immunohistochemistry experiment, 4 *μ*m thick serial sections were made with a slicer and fixed on slides. Then, we performed immunohistochemistry experiments according to the manufacturer's protocol. Each specimen was incubated with monoclonal antibodies against human FBXW7 (1 : 200; Abcam, USA) and BGN (1 : 200; CST, USA) overnight at 4°C. After eluting the primary antibody, the secondary antibody was routinely incubated for 50 minutes. Immunostaining was performed using DAB reagent, and the cell nucleus was counterstained. Finally, the samples were dehydrated with anhydrous ethanol and sealed with xylene transparent neutral gum. We scanned and analyzed the samples with an automatic digital slide scanning and analysis system (Aperio VERSA 8, Germany) and used the immune response score (IRS) method to semiquantitatively score the gene expression levels [26].

IF: we prepare cell sliders and perform IF experiments according to the merchant's instructions. Antibody against human HMGB1 (1 : 200, Proteintech, USA) was used. We scanned and analyzed the samples with an automatic digital slide scanning and analysis system (Aperio VERSA 8, Germany).

### 2.3. Cell Line Culture

The cell lines involved in this study included five GC cell lines (AGS, HGC-27, MGC-803, MKN-45, and SGC-7901) and a human stomach epithelial cell line (GES-1), which were obtained from the Cell Bank of Wuhan University. All cells were incubated in an incubator with 5% CO_2_ at 37°C using DMEM (Gibco, USA) containing 10% fetal bovine serum (FBS) (Gibco, USA) and 2 mmol/L glutamine. When the cell density reached more than 90%, 0.25% trypsin was used to isolate the cells for subculture and subsequent experiments.

### 2.4. RNA Extraction and Quantitative Reverse Transcription Polymerase Chain Reaction (qRT-PCR) Assay

Total RNA was extracted from the GC cell lines and tissue samples by using the EASYspin Tissue and Cellular RNA Rapid Extraction Kit (Aidlab, Beijing, China) or TRIzol Reagent (Invitrogen, USA) according to the manufacturer's protocol. Then, the concentration of total RNA was measured by a NanoDrop 2000 full-spectrum spectrophotometer (Thermo Scientific, USA), and 1 *μ*g of RNA from each sample was reverse-transcribed into cDNA according to the related kit's instructions (Vazyme, Nanjing, China). Next, we performed qRT-PCR experiments on a Bio-Rad IQ5 real-time PCR instrument (Bio-Rad, USA) by using SYBR-Green PCR Master Mix (Vazyme, Nanjing, China) in a 20 *μ*L reaction system. The 2^-∆∆Ct^ method was used to calculate the relative expression of the target gene. The gene primer sequences involved in this study are shown in Supplementary Table 1. Finally, the histogram was plotted using GraphPad Prism 8.

### 2.5. Subcellular Fractionation Assay for Localization Studies and RNA Fluorescence In Situ Hybridization (FISH) Assay

GC cells (1 × 10^7^) were collected and washed twice with ice-cold PBS. According to the manufacturer's protocol, we carried out nuclear and cytosolic fractionation assays using the Nuclear and Cytoplasmic Extraction Kit. Then, total RNA was extracted from the nuclear fraction and cytoplasmic fraction samples by using the RNA Rapid Extraction Kit (Aidlab, Beijing, China). Finally, the relative expression of SEMA3B-AS1 in the nucleus and cytoplasm was detected by qRT-PCR assay, and U6 and *β*-actin were used as nuclear and cytoplasmic reference genes, respectively.

The FISH probe of SEMA3B-AS1 was synthesized by GenePharma (Shanghai, China). The probe sequence is shown in Supplementary Table 1. First, we prepared SGC and HGC cells into cell climbing slides and 4% paraformaldehyde-fixed SGC and HGC cell slides. Then, FISH experiments were conducted according to the FISH kit protocol (Servicebio, Wuhan). In brief, we treated the cells with protease K (20 *μ*g/mL) for 1-55 minutes and washed them with PBS three times. Next, we performed prehybridization, hybridization (probe hybridization solution at a concentration of 5 *μ*M), and DAPI counterstaining of the nuclei. Finally, we observed the results, captured images under a positive fluorescence microscope (NIKON, Japan), and analyzed the results.

### 2.6. Lentivirus, Plasmids, siRNAs, and Transfection

The construction, synthesis, sequencing, and identification of the plasmid and lentivirus of SEMA3B-AS1 containing green fluorescent protein (GFP) were completed by GenePharma Biotechnology Co. Ltd. The plasmid and lentivirus of BGN, LV-OE-FBXW7/LV-ON-FBXW7, HMGB1-RNAi, FBXW7-RNAi (1, 2, 3), and negative control RNA (si-control) were purchased from GeneChem Chemical Technology Co. Ltd. (Shanghai, China). Double luciferase-associated plasmids and HMGB1 overexpression plasmid were purchased from BIOFAVOR BIOTECH. GC cells (SGC-7901, HGC-27) were transfected using Lipofectamine 2000 (Invitrogen, USA) according to the manufacturer's protocol. At 24 or 48 h after transfection, the cells were plated for functional assay or harvested for RNA or protein correlation analysis, respectively. To construct stably transfected cell lines, lentivirus and polybrene (final 7 *μ*g/mL, Sigma-Aldrich) were added to 25% of the confluent cells, fresh DMEM containing 10% FBS was added 16 h after infection, and the medium was exchanged with medium containing the corresponding antibiotics 48 h later. Finally, stably transfected cell lines were obtained by further screening with puromycin (Sigma-Aldrich, USA) for subsequent tests.

### 2.7. Protein Extraction, Western Blotting Assay, and Reagents

Cultured GC cells were collected and washed three times with PBS, and then, RIPA buffer containing protease inhibitor (Thermo Scientific, USA) was added to the cells according to the number of cells. The cell lysates were dissolved on ice for 30 minutes and then ultrasonically crushed and quantified by using BCA assays. The proteins were separated by sodium dodecyl sulfate-polyacrylamide gels (SDS-PAGE) and transferred to polyvinylidene fluoride (PVDF) membranes (Millipore, USA). Next, the membranes were blocked with 5% nonfat milk for 2 h, and the primary antibodies were incubated on a shaker in a refrigerator at 4°C overnight. Then, the membranes were washed with TBST, and HRP-conjugated secondary antibodies were added for 1-1.5 h at room temperature. The target proteins were detected with a Bio-Rad ChemiDoc XRS System. Densitometric analysis was performed by using Bio-Rad Image Lab software. The primary antibodies and dilution multiples involved in this study were as follows: anti-FBXW7 (1 : 1000, Abcam, USA), anti-BGN (1 : 1000, CST, USA), anti-HMGB1 (1 : 1000, Proteintech, USA), anti-H3 (1 : 1000, CST, USA), and anti-GAPDH (1 : 5000, Santa Cruz, CA). The secondary antibodies were diluted at 1 : 5000.

### 2.8. Cell Counting Kit-8 (CCK-8) Assay, Colony Formation Assay, and Wound Healing Assay

To detect the changes in cell viability after overexpression or knockdown of SEMA3B -AS1, we performed a CCK-8 assay according to the manufacturer's instructions (Shanghai, China), detected the absorbance of cells at 0 h, 24 h, 48 h, 72 h, and 96 h, and analyzed the differences in absorbance between each group.

A colony formation experiment was carried out to detect the proliferation ability of cells after SEMA3B-AS1 overexpression or interference. In short, SGC-7901 cells and HGC cells were implanted into a six-well plate at 1000 cells per well. After 14 days of culture in an incubator at 37°C, the samples were fixed with 4% paraformaldehyde, stained with 0.5% crystal violet, and then photographed under a microscope (Olympus-IX73, Japan).

A wound healing assay was performed to evaluate the migration ability of GC cells after SEMA3B-AS1 overexpression or knockdown. GC cells were plated into six-well plates, and when the cell density was approximately 90%, three wounds were created using a plastic pipette tip on the cell surface, and the cellular debris was washed away with PBS. Next, we added serum-free medium and incubated the cells in an incubator at 37°C. Photos of the same area were taken again every 24 h. Finally, a microscope (Olympus-IX73, Japan) was utilized to take photographs, and the wound area was measured by ImageJ software (NIH, USA). Each experiment was repeated at least three times.

### 2.9. Transwell Migration and Invasion Assay

We used 24-well plates (8 *μ*m pore size; Corning, USA) to detect the migration and invasion ability of GC cells after SEMA3B-AS1 overexpression or knockdown. A total of 1.0 × 10^5^ cells were suspended in 500 *μ*L serum-free DMEM and added to the upper chamber, while 750 *μ*L DMEM containing 10% FBS was added to the lower chamber. After incubation at 37°C for 48 h, we removed the cells in the upper chamber, and cells on the lower surface of the membrane were fixed with 4% paraformaldehyde and stained with crystal violet (0.5%). Finally, five visual fields were selected under a microscope (Olympus-IX73, Japan) to take photos and count cells. All experiments were repeated three times.

### 2.10. RNA Pull-Down Assay

Based on the gene sequence of SEMA3B-AS1, we designed and synthesized a biotin-labeled full-length RNA probe from RiboBio Co. Ltd. (Guangzhou, China).RNA pull-down assays were performed in SEMA3B-AS1-overexpressing cells (HGC-27) using the RNA-Protein Pull-Down Kit (Thermo 20164, USA). In brief, biotinylated full-length RNA probes were mixed with streptavidin agarose beads and incubated overnight at 4°C. A freshly prepared reagent was then added to each reaction system with RNase inhibitor and protease/phosphatase inhibitor. The mixture was incubated with rotation for 2 h at room temperature. After washing thoroughly three times, the RNA-protein binding mixture was eluted, and the eluted products were identified through Western blotting or mass spectrometry (MS).

### 2.11. RNA-Binding Protein Immunoprecipitation (RIP) Assay

To explore the correlation between lncRNAs and proteins, we performed a RIP assay. GC cells (HGC-27 or SGC-7901) were cultured and treated accordingly. Next, the cells were harvested and lysed with RIP lysis buffer. Then, we carried out the following experiment according to the protocol of the Magna RIP Kit (Millipore, Billerica, MA, USA). Anti-HMGB1 (5 *μ*g, Proteintech, USA) and anti-IgG (2 *μ*g) antibodies were added and incubated at room temperature for 30 minutes, and total RNA (10 *μ*L, input control) was utilized as a control. Finally, RNA was extracted and purified by the TRIzol method, and the RNA concentration was detected by a NanoDrop 2000 full-spectrum spectrophotometer. Next, the RNAs were reverse transcribed to cDNA and measured by qRT-PCR assay.

### 2.12. Chromatin Isolation by RNA Purification (ChIRP) Assay

To explore the correlations between lncRNAs and proteins, we performed a ChIRP assay. According to the gene sequence of SEMA3B-AS1, a biotin-labeled ChIRP probe was designed by us and synthesized by RiboBio Co. Ltd. (Guangzhou, China). Then, we performed a ChIRP assay according to a reference book and the protocol of the ChIRP assay kit (Guangzhou, China). The biotin probe sequences of SEMA3B-AS1 are listed in Supplementary Table 1. The protein product of the ChIRP experiment was detected by Western blotting.

### 2.13. Dual-Luciferase Reporter Assay

We first cloned the FBXW7 promoter into the PGL4.17 basic luciferase reporter vector (Promega, USA), and SEMA3B-AS1 and HMGB1 were cloned into the pCDNA3.1 plasmid. In brief, HEK-293T cells (1 × 10^4^) were seeded into 96-well plates and cultured for 24 h. Then, related plasmids were cotransfected according to the protocol and experimental groups. Each well was transfected with 5 ng Renilla reporter plasmid, 100 ng expression vector (PGL4.27-HMGB1 or PGL3-BASIC), and FBXW7 promoter plasmid, which were normalized after cotransfection. After 24 h of incubation, the luciferase activity of PGL4.17-FBXW7-Pro was detected using a Dual-Luciferase Reporter Gene Analysis System (Promega, USA).

### 2.14. Coimmunoprecipitation (co-IP) Assay

According to the protocol of the co-IP kit (Abison, Guangzhou, China), the cells needed for the experiment were cultured and collected. After removing the DMEM, the cells were washed once with 1x PBS. Then, 0.5 mL of ice-cold lysis buffer was added to the cell plate (10 cm) and incubated on ice for at least 5 minutes. Next, we scraped off the cells and transferred them to a new EP tube. Then, the follow-up experiment was completed according to the operation process of the kit. Finally, an immunoprecipitation- (IP-) immunoblotting (IB) test was carried out to detect and analyze the binding of the FBXW7 and BGN proteins.

### 2.15. Animal Experiment

Six- to eight-week-old female nude mice (BALB/c) were purchased from Jiangsu Jicui Yaokang Biotechnology Co. Ltd. (Nanjing, China). To establish a subcutaneous tumorigenesis model in nude mice, we randomly divided the mice into three groups (OeNC-SEMA3B-AS1-HGC, Oe-SEMA3B-AS1-HGC, and Oe-SEMA3B-AS1-HGC+SH-FBXW7) (*n* = 5 mice/group). A total of 5 × 10^5^ GC cells (HGC-27) were suspended in 0.1 mL DMEM and injected into the flank of each mouse. After two weeks, we began to observe subcutaneous tumor formation and assess tumor volume once a week, tumor volume = 1/2 × (width^2^ × length). At the 5th week or when the tumor diameter was over 1.5 cm, the mice were euthanized, and the tumors were removed for subsequent experiments. For the intra-abdominal tumor model, we randomly divided the mice into three groups (OeNC-SEMA3B-AS1-HGC, Oe-SEMA3B-AS1-HGC, and Oe-SEMA3B-AS1-HGC+SH-FBXW7) (*n* = 6 mice/group). A total of 1 × 10^6^ GC cells (HGC-27) were suspended in 0.15 mL DMEM, and then, the right lower abdominal cavity was selected for injection. Five weeks later, the mice were euthanized. Then, we dissected the mice and evaluated abdominal tumor formation and tumor load. Finally, we assessed the expression of the involved genes in tumor tissues by immunohistochemical staining.

### 2.16. Statistical Analysis

We used SPSS software (version 23.0, IBM SPSS, USA) and GraphPad Prism (version 8.0, GraphPad Software, USA) to analyze all the data in this study. To evaluate the relationship between SEMA3B-AS1 expression and BGN expression, Pearson's correlation analysis was performed. Then, we utilized the chi-square test to analyze the expression of SEMA3B-AS1 and BGN and the clinicopathological status of GC patients. Two groups were compared using Student's *t*-test, Wilcoxon test, or Fisher's exact test. Comparisons between groups were performed by variance analysis or the chi-square test. Moreover, the Kaplan-Meier survival curve and log-rank test were applied for survival analysis. Finally, the independent factors affecting the prognosis of gastric cancer were determined by using univariate and multivariate Cox regression analyses. All cell experiments were carried out independently at least three times. In all cases, the results were considered statistically significant when the *p* value < 0.05.

## 3. Results

### 3.1. SEMA3B-AS1 Is Downregulated in Human GC Tissues and Mainly Located in the Nucleus

**Figure 1:**
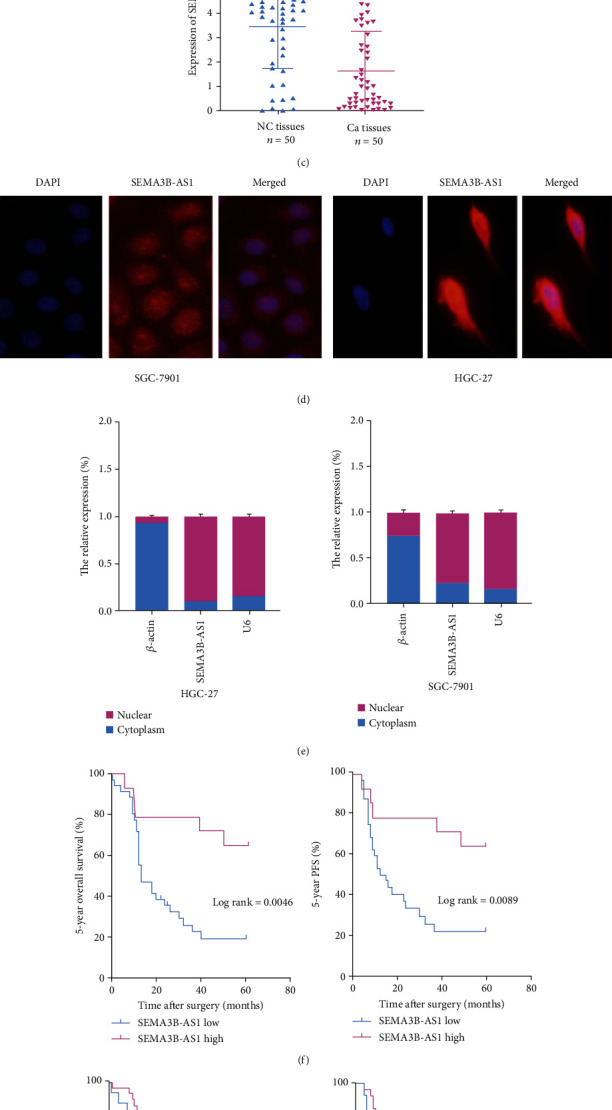
SEMA3B-AS1 was downregulated in human GC tissues. It was mainly located in the nucleus. Data are shown as mean ± SEM (*n* = 3); ^∗^*p* < 0.05, ^∗∗^*p* < 0.01, ^∗∗∗^*p* < 0.001, and ^∗∗∗∗^*p* < 0.0001. (a) lncRNA expression was identified in three pairs of GC tissues and corresponding PM of GC tissues by microarray analysis according to the value of the samples and fluorescence intensity. (b) The relative mRNA expression of SEMA3B-AS1 in five GC cell lines and normal stomach epithelial cell line (GES-1) was identified by qRT-PCR assay. (c) The relative mRNA expression of SEMA3B-AS1 was identified by qRT-PCR assay in 50 pairs GC tissues and adjacent normal tissues. (d) The intracellular location of SEMA3B-AS1 was identified by RNA FISH assay (magnification: ×400). (e) The nuclear and cytoplasmic distribution of SEMA3B-AS1 in HGC-27 and SGC-7901 cell lines was identified using qRT-PCR assay. (f) The correlation with the expression level of SEMA3B-AS1 between five-year overall survival (OS) and five-year progression-free survival (PFS) was analyzed through Kaplan-Meier survival analysis. (g) The correlation with (PM) between five-year overall survival (OS) and five-year progression-free survival (PFS) was analyzed through Kaplan-Meier survival analysis.

According to the lncRNA sequencing results of our research group, we found that SEMA3B-AS1 expression was decreased in PM tissues compared to primary lesion tissues (Figure 1(a)). Next, we verified the SEMA3B-AS1 mRNA expression level in five gastric cancer cell lines (AGS, HGC-27, MGC-803, MKN-45, and SGC-7901) and a normal stomach mucosal cell line (GES-1) through qRT-PCR assay, and it was also demonstrated that SEMA3B-AS1 expression was decreased in GC cells compared to GES-1 cells (Figure 1(b)). Moreover, we detected the mRNA expression level of SEMA3B-AS1 in 50 pairs of GC tissues and corresponding adjacent normal tissues using qRT-PCR assays. The relative mRNA expression of SEMA3B-AS1 was significantly decreased in GC tissues compared with normal tissues, and the difference was statistically significant (*p* < 0.0001) (Figure 1(c)). The function of lncRNAs is closely related to their localization. To determine the localization of SEMA3B-AS1, FISH assays and subcellular fractionation assays (for localization assessment) were carried out in SGC-7901 or HGC-27 cells, and we found that SEMA3B-AS1 was mainly localized in the nucleus (Figures 1(d) and 1(e)). These data demonstrated that SEMA3B-AS1 was mainly localized in the nucleus and downregulated in human GC.

### 3.2. SEMA3B-AS1 Is Associated with Metastasis and Prognosis in GC Patients

Based on the clinical data of 50 GC patients, we explored the correlations between clinicopathological features and SEMA3B-AS1 expression levels (Table 1). The results showed that the low expression of SEMA3B-AS1 was obviously related to tumor size, T stage, lymphatic metastasis, distant metastasis, tumor-node-metastasis (TNM) stage, and PM (*p* < 0.05). However, it was not associated with sex, age, cancer grade, CEA, or CA19-9 (*p* > 0.05). Moreover, the survival analysis demonstrated that patients with low SEMA3B-AS1 expression had much shorter 5-year overall survival (OS) (Figure 1(f)) and 5-year progression-free survival (PFS) (Figure 1(f)) times than those who had high SEMA3B-AS1 expression. The 5-year overall survival rate analysis showed that the survival rate of patients with low SEMA3B-AS1 expression in cancer tissues was significantly lower than that of patients with high SEMA3B-AS1 expression in cancer tissues (9/27 (33.33%) vs. 9/14 (64.29%) *p* < 0.05). Univariate and multivariate analyses revealed that SEMA3B-AS1 expression was obviously related to poor PFS (HR: 0.123, 95% CI: 0.023–0.662, *p* = 0.015) and OS (HR: 0.113, 95% CI: 0.021–0.603, *p* = 0.011) (Table 2). These results demonstrated that SEMA3B-AS1 is significantly correlated with the progression and metastasis of GC.

### 3.3. SEMA3B-AS1 Inhibits the Activity, Invasion, Proliferation, and Migration and EMT of GC Cells In Vitro

**Figure 2:**
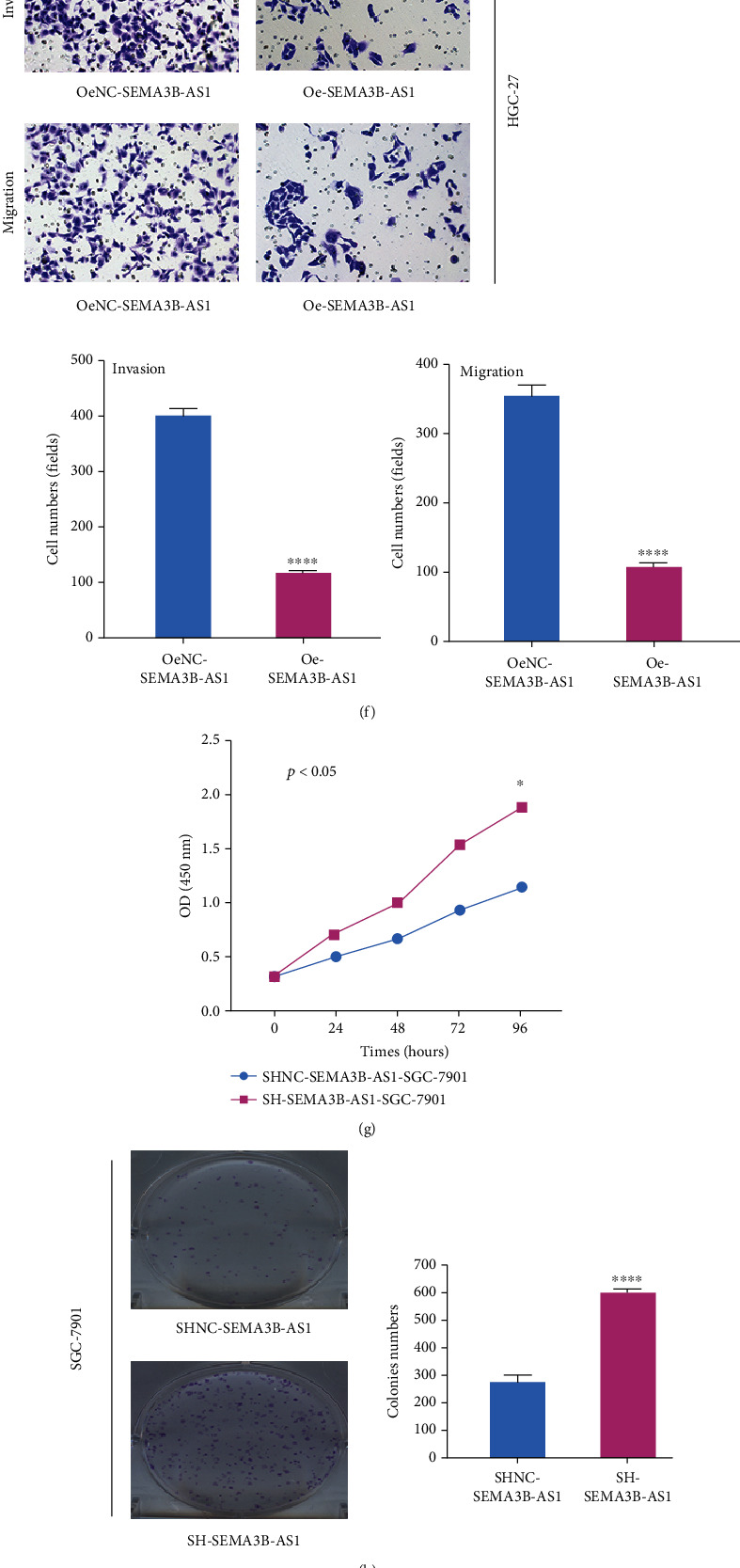
SEMA3B-AS1 could inhibit the activity, invasion, proliferation, and migration and EMT ability of GC cells in vitro (magnification: ×200). Data were shown as mean ± SEM (*n* = 3); ^∗^*p* < 0.05, ^∗∗^*p* < 0.01, ^∗∗∗^*p* < 0.001, and ^∗∗∗∗^*p* < 0.0001. (a) The overexpression efficiency of Lv-Oe-SEMA3B-AS1 and negative control (NC) was detected in HGC-27 using qRT-PCR. (b) The expression efficiency of Lv-SH-SEMA3B-AS1 and negative control (NC) was detected in SGC-7901 using qRT-PCR. (c) The viability of HGC-27 cells after SEMA3B-AS1 overexpression and negative control (NC) was detected by CCK-8 assay. (d-f) The cell proliferation, migration and invasion ability of GC cells (HGC-27) negative control and SEMA3B-AS1 overexpression were detected by the colony formation assay (d), wound healing assay (e), and transwell assay (f), respectively. (g) The viability of SGC-7901 cells after SEMA3B-AS1 knockdown and negative control (NC) was detected by CCK-8 assay. (h-j) The cell proliferation,migration , and invasion ability of GC cells (SGC-7901) negative control and SEMA3B-AS1 knockdown were detected by the colony formation assay (h), wound healing assay (i), and transwell assay (J), respectively. (k) The expression of EMT markers (E-cadherin and vimentin) in GC cells (HGC-27) was analyzed by Western blotting assay after SEMA3B-AS1 overexpression and negative control. (l) The expression of EMT markers (E-cadherin and vimentin) in GC cells (SGC-7901) was analyzed by Western blotting assay after SEMA3B-AS1 knockdown and negative control.

Given the important role of SEMA3B-AS1 in metastasis, OS, and PFS of GC patients, gain- or loss-of-function tests were performed to verify the biological functions of SEMA3B-AS1. According to the expression characteristics of SEMA3B-AS1 in GC cell lines, SGC-7901 was selected as the knockdown model cell line, and HGC-27 was selected as the overexpression model cell line (Figure 1(b)). Then, stable cell lines were constructed and named LV-SH-SEMA3B-AS1-SGC and Lv-Oe-SEMA3B-AS1-HGC. Next, we detected the efficiency of overexpression and knockdown of SEMA3B-AS1 through qRT-PCR assay (Figures 2(a) and 2(b)).

The viability, proliferation, invasion, and migration of GC cells were detected by CCK-8 assay, colony formation assay, transwell assay, and wound healing assay, respectively, after SEMA3B-AS1 overexpression. The results showed that the viability (Figure 2(c)), proliferation (Figure 2(d)), migration (Figure 2(e)), and invasion (Figure 2(f)) of SEMA3B-AS1-overexpressing cells were obviously decreased compared with those of the negative control group. In contrast, after knockdown of SEMA3B-AS1 in SGC cells, the viability (Figure 2(g)), proliferation (Figure 2(h)), migration (Figure 2(i)), and invasion (Figure 2(j)) abilities of GC cells were increased. Previous experimental results showed that SEMA3B-AS1 was significantly associated with lymph node metastasis and PM in patients with GC. We speculated that SEMA3B-AS1 might be closely related to the EMT ability of tumor cells. Thus, we detected EMT indicators, including vimentin and E-cadherin, in HGC-27 or SGC-7901 cells after SEMA3B-AS1 overexpression or knockdown using Western blotting. The results demonstrated that the protein expression of E-cadherin was increased, while that of vimentin was decreased after SEMA3B-AS1 overexpression (Figure 2(k)). However, in SEMA3B-AS1 knockdown cells, we obtained the exact opposite results (Figure 2(l)). These data demonstrated that the overexpression of SEMA3B-AS1 could decrease the viability, invasion, proliferation, migration, and EMT of GC cells.

### 3.4. SEMA3B-AS1 Can Combine with HMGB1

**Figure 3:**
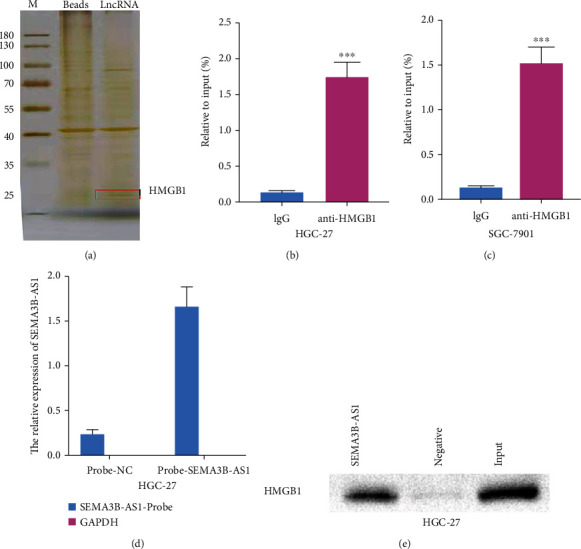
SEMA3B-AS1 can combine with HMGB1. Data are shown as mean ± SEM (*n* = 3); ^∗^*p* < 0.05, ^∗∗^*p* < 0.01, ^∗∗∗^*p* < 0.001, and ^∗∗∗∗^*p* < 0.0001. (a) RNA pull-down assay showed that biotinylated SEMA3B-AS1 could bind with HMGB1 in GC cells (HGC-27) in vitro. RNA immunoprecipitation (RIP) assay demonstrated the direct binding relationship between SEMA3B-AS1 and HMGB1 in (b) HGC-27 and (c) SGC-7901 cells; the RIP RNA products were detected by qRT-PCR for SEMA3B-AS1. The fold enrichment of SEMA3B-AS1 was relative to its corresponding IgG control. (d) The enrichment of SEMA3B-AS1 in the probe group targeting SEMA3B-AS1 relative to NC probe in HGC-27 cells, as detected by ChIRP assay. GAPDH served as a negative control (NC). (e) The enrichment of HMGB1 protein in the probe group targeting SEMA3B-AS1 relative to the NC probe in HGC-27 cells, as detected by ChIRP assay and the protein product was identified through Western blotting.

Based on a previous study, we demonstrated that SEMA3B-AS1 plays a vital role in GC progression and metastasis, but the exact mechanism is unclear. Existing research has demonstrated that lncRNAs bind to DNA, RNA, and proteins to regulate gene expression via multiple mechanisms, including epigenetic regulation, transcriptional regulation, and posttranscriptional regulation [27]. To explore the mechanism of SEMA3B-AS1, we conducted a biotin RNA pull-down experiment to identify potential proteins binding to SEMA3B-AS1 (Figure 3(a)). By MS analysis, we found that HMGB1, a multifunctional molecule with different roles at different sites, was an interacting protein of SEMA3B-AS1 (Supplementary Table 2, RNA pull-down-MS). To further confirm the mutual binding of SEMA3B-AS1 and HMGB1, we conducted RIP experiment. We found significant enrichment of HMGB1 with the anti-HMGB1 antibody versus IgG in HGC-27 or SGC-7901 GC cells (Figures 3(b) and 3(c)). Moreover, we demonstrated the interaction between SEMA3B-AS1 and HMGB1 using a ChIRP assay. We found significant enrichment of HMGB1 in the probe group targeting SEMA3B-AS1 relative to the negative control probe by testing the protein product of the ChIRP experiment through Western blotting (Figures 3(d) and 3(e)). These data revealed that SEMA3B-AS1 could bind with the HMGB1 protein.

### 3.5. SEMA3B-AS1 Promotes the Expression of FBXW7 by Binding HMGB1

**Figure 4:**
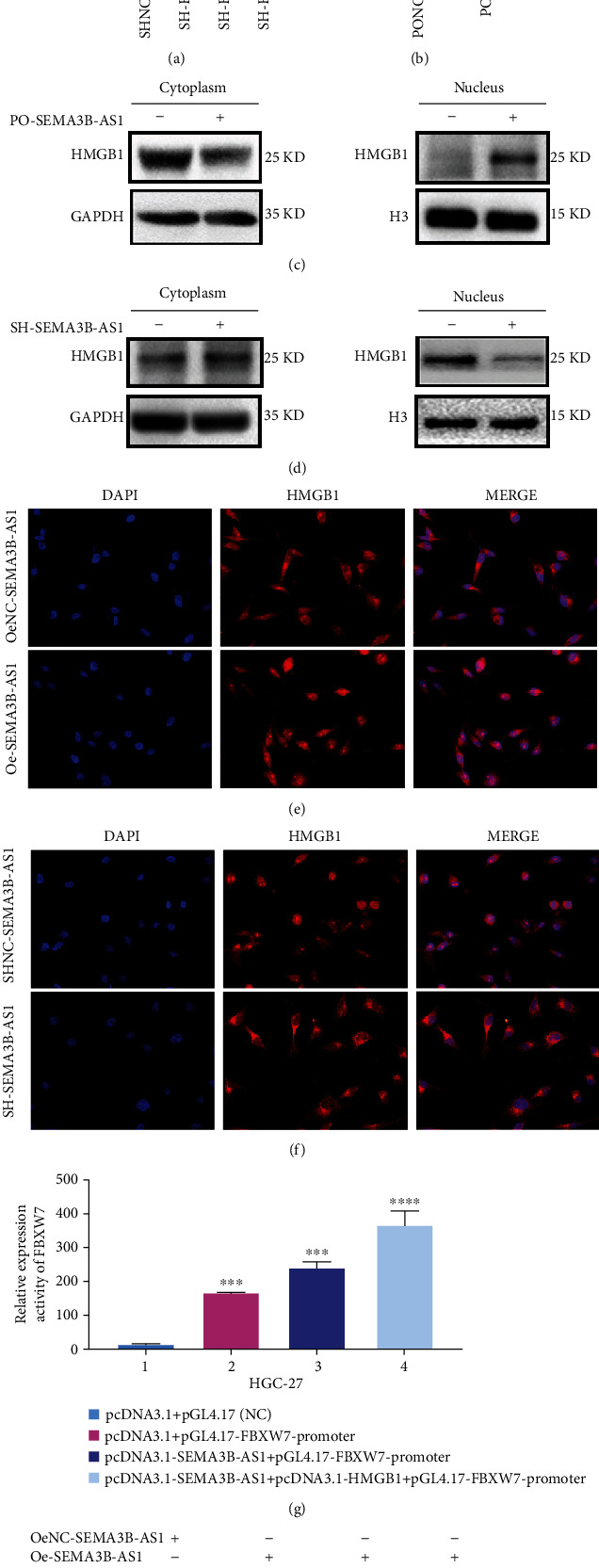
SEMA3B-AS1 promotes the expression of FBXW7 by binding HMGB1. Data are shown as mean ± SEM (*n* = 3); ^∗^*p* < 0.05, ^∗∗^*p* < 0.01, ^∗∗∗^*p* < 0.001, and ^∗∗∗∗^*p* < 0.0001. (a) The efficiency of HMGB1 knockdown was verified in SGC cells through qRT-PCR assay. (b) The efficiency of HMGB1 overexpression was verified in HGC cells through qRT-PCR assay. The expression levels of HMGB1 protein in the nucleus and cytoplasm of SEMA3B-AS1 (c) overexpressed or (d) interfered cells were detected by Western blotting. (e) The localization of HMGB1 was analyzed by immunofluorescence when SEMA3B-AS1 was overexpressed. Red fluorescence represents HMGB1. (f) The localization of HMGB1 was analyzed by immunofluorescence when SEMA3B-AS1 was knocked down. Red fluorescence represents HMGB1. (g) Dual-Luciferase Assays to assess FBXW7 promoter activity while SEMA3B-AS1 was either alone or in combination with HMGB1. (h) The changes of FBXW7 protein were detected by Western blotting after overexpression or interference of HMGB1 in SEMA3B-AS1-overexpressed gastric cancer cells.

In our previous study, FBXW7 was found to play a crucial role in the occurrence and development of GC patients, and overexpression of FBXW7 inhibited the progression of GC [19, 28]. Therefore, we continued to explore whether SEMA3B-AS1 combined with HMGB1 could regulate the expression of FBXW7 to further clarify the regulatory mechanism of FBXW7 in GC. First, we constructed HMGB1 overexpression and interference plasmids and measured the efficiency of interference and overexpression in SGC and HGC cells, respectively (Figures 4(a) and 4(b)). Then, we used Western blotting assay to detect HMGB1 protein changes in cytoplasm and nucleus in the SEMA3B-AS1 overexpressed or interfered GC cells; the results showed that the HMGB1 protein in the nucleus was significantly increased, when SEMA3B-AS1 was overexpressed, while SEMA3B-AS1 was knocked down, the HMGB1 protein in the nucleus was significantly decreased (Figures 4(c) and 4(d)). Next, since the biological function of HMGB1 is related to its localization, we needed to identify the localization status of HMGB1 in gastric cancer cells when SEMA3B-AS1 was overexpressed or knocked down. Localization analysis of HMGB1 by immunofluorescence showed that HMGB1 was mainly located in the nucleus when SEMA3B-AS1 was overexpressed (Figure 4(e)). Yet when SEMA3B-AS1 was knocked down, HMGB1 was displaced mainly in the cytoplasm (Figure 4(f)). To further confirm that HMGB1 can regulate the transcription of FBXW7 gene, we cloned the FBXW7 promoter into the PGL4.17 basic luciferase reporter vector and cloned SEMA3B-AS1 and HMGB1 into the pCDNA3.1 plasmid. Next, the luciferase activity of PGL4.17-FBXW7-Pro was detected using a Dual-Luciferase Reporter Gene Analysis System. The results demonstrated that when SEMA3B-AS1 was combined with HMGB1, the transcription of FBXW7 was significantly enhanced, and the difference between them was statistically significant (*p* < 0.05) (Figure 4(g)). Moreover, the changes in FBXW7 protein were detected by Western blotting assay after overexpression of SEMA3B-AS1 or combined overexpression and interference of HMGB1. The results showed that the protein level of FBXW7 was greatly increased after combined overexpression of SEMA3B-AS1 and HMGB1 (Figure 4(h)). These data demonstrated that SEMA3B-AS1 could facilitate FBXW7 expression by combining with HMGB1.

### 3.6. SEMA3B-AS1 Might Destabilize the BGN Protein by Regulating FBXW7, Thus Promoting the Ubiquitination Degradation of BGN and Inhibiting GC

**Figure 5:**
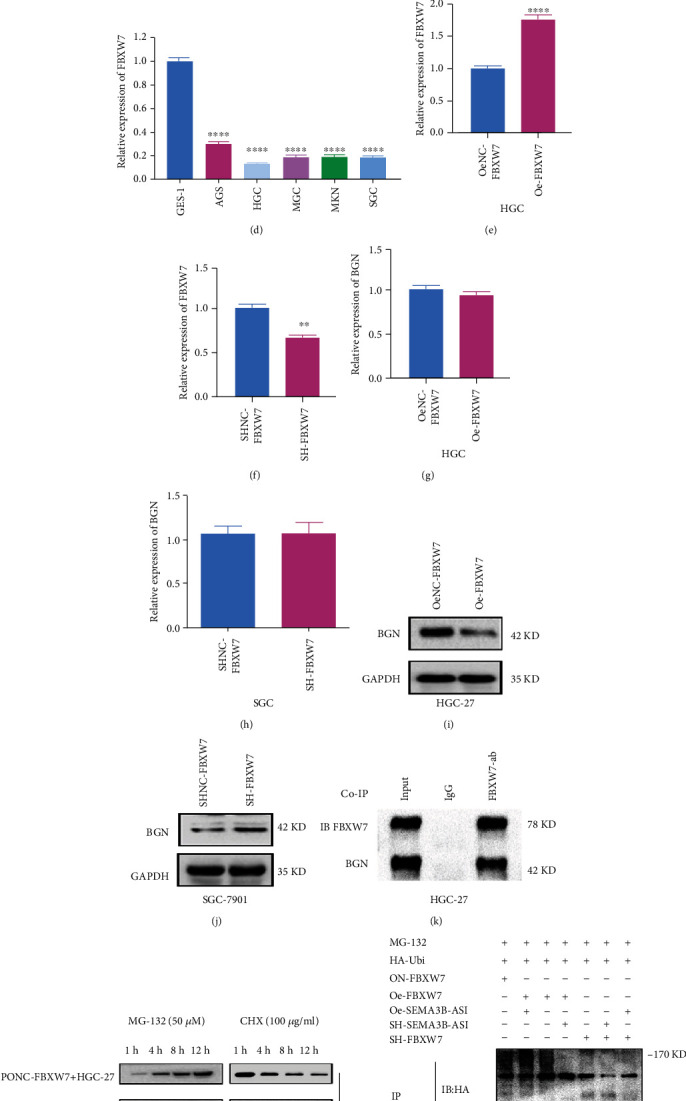
SEMA3B-AS1 might destabilize the BGN protein by regulating FBXW7, thus promoting the ubiquitination degradation of BGN and inhibiting GC. Data were shown as mean ± SEM (*n* = 3); ^∗^*p* < 0.05, ^∗∗^*p* < 0.01, ^∗∗∗^*p* < 0.001, and ^∗∗∗∗^*p* < 0.0001. (a) The expression of BGN protein in gastric cancer and adjacent normal tissues was analyzed by bioinformatics using GSE54129 data set. (b) The relative mRNA expression of BGN was identified by qRT-PCR assay in 50 pairs of GC tissues and adjacent normal tissues. (c) The representative IHC staining image for BGN in the paracancerous normal tissues, GC tissues, and GS PM tissues. (d) The relative mRNA expression of FBXW7 in five GC cell lines and normal stomach epithelial cell line (GES-1) was detected by qRT-PCR assay. (e) The overexpression efficiency of FBXW7 and negative control (NC) was detected in HGC-27 using qRT-PCR. (f) The knockdown efficiency of FBXW7 and negative control (NC) was detected in SGC-7901 using qRT-PCR. The mRNA expression levels of BGN were detected by qRT-PCR assay after FBXW7 (g) overexpression or (h) knockdown in GC cells (HGC-27 or SGC-7901). The protein expression levels of BGN were detected by Western blotting assay after FBXW7 (i) overexpression or (j) knockdown in GC cells (HGC-27 or SGC-7901). (k) The interaction between FBXW7 and BGN was identified in HGC-27 cells by coimmunoprecipitation (co-IP). SDS-PAGE separated the immunoprecipitates of input (20%) and BGN. Western blotting was performed to confirm the interaction between FBXW7 and BGN. (l) Cell groups were treated with the proteasome inhibitor MG-132 (50 *μ*M) or the protein synthesis inhibitor cycloheximide CHX (100 *μ*g/mL) at different time points (1 h, 4 h, 8 h, and 12 h), and the changes of BGN protein at different time points were detected by Western blotting assay. (m) HGC-27 or SGC-7901 cells were treated with MG-132 (50 *μ*M) and transfected with HA-Ubi and simultaneously overexpression or knockdown FBXW7 as well as SEMA3B-AS1 overexpressed or knocked down, respectively, FBXW7 vector served as a negative control. Cell lysates were immunoprecipitated with anti-BGN antibody to identify ubiquitination of BGN with anti-HA antibodies using Western blotting assay. (n) The mRNA relative expression levels of SEMA3B-AS1 and BGN were obvious negative association in GC patients using Pearson's correlation analysis (Rs = −0.5568, *p* < 0.0001).

We found that BGN protein was significantly overexpressed in PM tissues by MS analysis of primary tumors and GC PM samples. Then, the expression of BGN protein in GC tissues and adjacent normal tissues was analyzed by bioinformatics using GSE54129 data set. The results showed that BGN protein was significantly overexpressed in GC tissues (*p* < 0.0001) (Figure 5(a)). Furthermore, BGN expression was detected in 50 pairs of GC and adjacent normal tissues, and the results also demonstrated that BGN was highly expressed in GC tissues compared to adjacent normal tissues (Figure 5(b)). Then, we used immunohistochemistry to detect BGN expression in normal adjacent tissues, GC tissues, and PM tissues. The results demonstrated that BGN expression was the highest in PM tissues (Figure 5(c)). Existing studies have shown that protein degradation is mainly affected by posttranscriptional modifications, including ubiquitination, glycosylation, and acetylation. We speculated that the BGN protein is degraded by ubiquitination. Then, we were surprised to find that the BGN protein is the ubiquitination substrate of FBXW7 through UbiBrowser website prediction. To verify our speculation, the mRNA expression of FBXW7 was detected by qRT-PCR in 5 gastric cancer cell lines (AGS, HGC, MGC, MKN, and SGC) and GES-1 (Figure 5(d)). According to the results, SGC was selected as the knockdown cell line, and HGC was selected as the overexpression cell line. The overexpression or knockdown efficiency of FBXW7 was verified through qRT-PCR assay (Figures 5(e) and 5(f)). To demonstrate that FBXW7 regulates BGN at the posttranscriptional level, BGN mRNA and protein expression levels were tested in FBXW7-overexpressing (HGC-27) or FBXW7 knockdown (SGC-7901) GC cells by qRT-PCR and Western blotting assays. The results showed that the BGN mRNA expression level was not significantly affected by FBXW7 overexpression or knockdown (Figures 5(g) and 5(h)), but the BGN protein level was significantly decreased or increased with FBXW7 overexpression or knockdown (Figures 5(i) and 5(j)). Next, we needed to prove the interaction between FBXW7 and BGN proteins, so a co-IP assay was performed, and the results demonstrated this fact (Figure 5(k)). Furthermore, to further test whether BGN is degraded by the ubiquitination pathway, we treated Lv-Oe-FBXW7-HGC-27 and Lv-OeNC-FBXW7-HGC-27 cells with cycloheximide (CHX) (a protein synthesis inhibitor) and MG-132 (a specific proteasome inhibitor). We found that MG-132 abolished the downregulation of BGN protein levels in FBXW7-overexpressing cells (Figure 5(l)). These results indicated that the ubiquitin-proteasome pathway might be required for the FBXW7-mediated degradation of BGN protein. To confirm that the ubiquitination level of BGN was affected by FBXW7, we conducted an IP assay and ubiquitination experiment after GC cell treatment with MG-132 (50 *μ*M), HA-Ubi plasmid transfection and FBXW7 overexpression or knockdown. The ubiquitination level of BGN was assessed with anti-HA Tag antibody. The ubiquitination level of BGN was increased in the FBXW7 overexpression group compared with the negative control group. However, the ubiquitination level of BGN was decreased after FBXW7 knockdown (Figure 5(m)). Moreover, we found a negative regulatory relationship between SEMA3B-AS1 and BGN by tissue validation of SEMA3B-AS1 and BGN (*p* < 0.0001) (Figure 5(n)). Further experiments have shown that when we overexpressed FBXW7 and SEMA3B-AS1 in HGC cells, the BGN ubiquitination level was the highest, and when we knocked down both factors in SGC cells, the BGN ubiquitination level was the lowest. However, when we simultaneously knocked down or overexpressed SEMA3B-AS1 in FBXW7-overexpressing or knockdown cells, the changes in BGN ubiquitination levels were partially reversed (Figure 5(m)). In conclusion, we found that FBXW7 could ubiquitylate the BGN protein, and its function was dependent on SEMA3B-AS1 to a large extent. At the same time, we could prove that the SEMA3B-AS1/HMGB1/FBXW7 axis exists.

### 3.7. Animal Experiments

**Figure 6:**
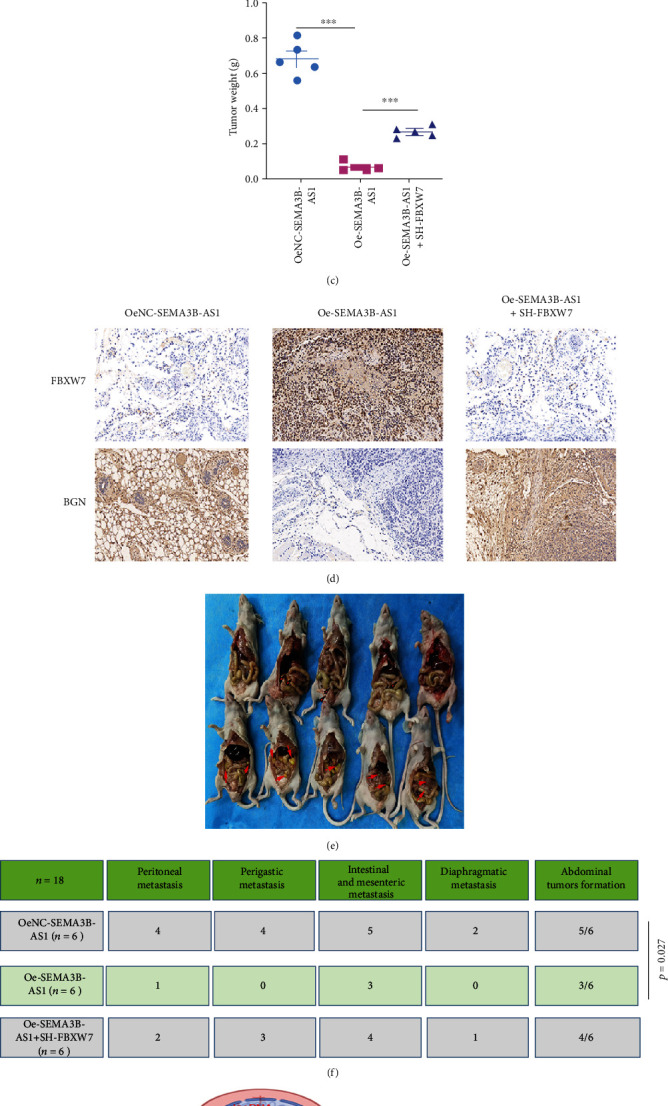
SEMA3B-AS1 could inhibit GC tumorigenesis and PM in vivo. Representative images of migratory or invaded cells (magnification, ×200) were shown. Data are shown as mean ± SEM (*n* = 3); ^∗^*p* < 0.05, ^∗∗^*p* < 0.01, and ^∗∗∗^*p* < 0.001. The morphological properties of (a) tumor subcutaneous xenograft, (b) tumor size, and (c) tumor weight in OeNC-SEMA3B-AS1-HGC-27, Oe-SEMA3B-AS1, and Oe-SEMA3B-AS1+SH-FBXW7-HGC-27 cells at 35 days, each group had five mice. (d) IHC analyzed the expression of FBXW7 and BGN protein of tumors from the OeNC-SEMA3B-AS1-HGC-27, Oe-SEMA3B-AS1, and Oe-SEMA3B-AS1+SH-FBXW7-HGC-27 cell groups. (e) The representative image of intraperitoneal tumor formation model from OeNC-SEMA3B-AS1-HGC-27 and Oe-SEMA3B-AS1-HGC-27. (f) Simultaneously, we counted and analyzed the number of peritoneal, perigastric, intestinal and mesenteric, and diaphragmatic metastases, each group had six mice. (g) The proposed mechanism model in which the SEMA3B-AS1/HMGB1/FBXW7 axis mediates the PM of GC by regulating BGN protein ubiquitination.

We further explored the role of SEMA3B-AS1 in the oncogenesis and metastasis of GC. We conducted animal experiments. For the subcutaneous xenograft model, six- to eight-week-old nude mice were randomly divided into three groups (*n* = 5), and OeNC-SEMA3B-AS1-HGC cells (5 × 10^5^), Oe-SEMA3B-AS1-HGC cells (5 × 10^5^), and Oe-SEMA3B-AS1+SH-FBXW7-HGC cells (5 × 10^5^) in 0.1 mL DMEM were injected into the flanks of mice. After 2 weeks, we began to measure the tumor size every week using digital vernier calipers and calculated the tumor volume using the following formula: volume = 1/2 × (width^2^ × length). At the fifth week of cell injection, the mice were euthanized, and subcutaneous tumors were removed. The results indicated that the volume and weight of tumors in the Oe-SEMA3B-AS1 group were significantly smaller than those in the OeNC-SEMA3B-AS1-HGC cell group and the Oe-SEMA3B-AS1+SH-FBXW7-HGC cell group (Figures 6(a)–6(c)). Furthermore, we performed an immunohistochemistry assay to identify the protein expression of FBXW7and BGN in the three groups of tumors. The results demonstrated that the expression of FBXW7 was significantly increased in the Oe-SEMA3B-AS1 group, while the BGN protein expression level was dramatically decreased (Figure 6(d)). For the intraperitoneal tumor formation model, Oe-SEMA3B-AS1-HGC cells (1 × 10^6^),Oe-SEMA3B-AS1-HGC cells (1 × 10^6^), and Oe-SEMA3B-AS1+SH-FBXW7-HGC cells (1 × 10^6^) were injected into the lower right abdominal cavity of each mouse. After 35 days, we euthanized the mice and dissected the abdominal cavity to assess tumor formation. We assessed abdominal invasion, including PM (1/6 mice), perigastric metastasis (0/6 mice), intestinal and mesenteric metastasis (3/6 mice), and diaphragmatic metastasis (0/6 mice), after SEMA3B-AS1 overexpression. However, in theOeNC-SEMA3B-AS1 group, we observed PM (4/6 mice), perigastric metastasis (4/6 mice), intestinal and mesenteric metastasis (5/6 mice), and diaphragmatic metastasis (2/6 mice). Statistical analysis of the two groups of data showed that the difference was statistically significant (*p* = 0.027). When we knocked down FBXW7 in the OE-SEMA3B-AS1 group, the tumor suppressive effect of SEMA3B-AS1 was partially attenuated (Figures 6(e) and 6(f)). These data demonstrated that SEMA3B-AS1 can inhibit GC oncogenesis in vivo. Taken together, these findings indicate that SEMA3B-AS1 can regulate GC formation and PM by regulating the BGN protein. The summary schematic of our findings from the current study is shown in Figure 6(g).

## 4. Discussion

Nuclear lncRNAs play a crucial role in many biological processes, and their dysregulation can lead to a variety of human cancers, including GC [29, 30]. Our present study is conducive to understanding the role of SEMA3B-AS1 downregulation in GC progression. Our data demonstrated that SEMA3B-AS1 overexpression could inhibit the PM of GC by regulating BGN protein ubiquitination. Some studies have found that SEMA3B-AS1 is an antioncogene in various cancers, including gastric cardia adenocarcinoma and esophageal squamous cell carcinoma [31, 32]. In these studies, SEMA3B-AS1 was confirmed to inhibit the progression of cardiac adenocarcinoma and to be closely related to the progression and prognosis of esophageal squamous cell carcinoma. Our data also confirmed that SEMA3B-AS1 plays an important role in GC progression and PM and is closely related to the survival and prognosis of GC patients.

HMGB1 plays a complex role in a variety of biological processes and has a variety of functions in maintaining cell homeostasis, and it is involved in the development of many diseases, such as inflammation and cancer. Recent studies have indicated that its function is closely related to its localization. It mainly assists in regulating telomeres and maintaining genomic stability in the nucleus, and its loss can lead to genomic instability and tumorigenesis [15, 16]. In the present study, we found that HMGB1 and SEMA3B-AS1 obviously interacted through RNA pull-down experiments when SEMA3B-AS1 was overexpressed. Moreover, we also demonstrated using RIP and ChIRP experiments that SEMA3B-AS1 can bind to HMGB1, thereby playing an important biological role. Furthermore, immunofluorescence localization revealed that HMGB1 was mainly located in the nucleus during SEMA3B-AS1 overexpression. However, after SEMA3B-AS1 interference, the HMGB1 was mainly located in the cytoplasm. Our experiments revealed that SEMA3B-AS1 can recruit and bind HMGB1 in the nucleus. In our previous studies, we demonstrated that FBXW7 can inhibit the progression and predict the prognosis of GC [19, 28]. In the current research, we found that the tumor suppressor gene FBXW7 is usually mutated and inactivated after the occurrence of cancer, and the body sends instructions for gene repair, which allows the antioncogene lncRNA to recruit and bind genes or proteins that can repair DNA damage. According to our data, we demonstrated that when SEMA3B-AS1 is overexpressed, HMGB1 is recruited. Then, SEMA3B-AS1 can promote the transcription and expression of FBXW7 through binding HMGB1, thus promoting autophagy and inhibiting apoptosis in tumor cells. In the absence of cancer-suppressive lncRNAs such as SEMA3B-AS1, HMGB1 cannot be recruited to play a vital role in DNA repair in time, but it may be transferred to the cytoplasm and extracellular space. The location of HMGB1 in the cytoplasm or extracellular space will promote the release of cytokines such as IL-6 and IL-8 by activating MAPK- and MyD88-dependent NF-KB pathways, thereby prompting tumor cell proliferation, angiogenesis, EMT, invasion, and metastasis. We revealed for the first time that SEMA3B-AS1 can bind HMGB1 to promote the transcription and expression of FBXW7, thus exerting its tumor suppressive effect.

In our analysis of MS results, we found that BGN protein expression was abnormally upregulated in PM samples. Recently, some studies have shown that abnormal expression of the BGN protein is closely associated with tumor metastasis and a poor prognosis for cancers, including GC and breast cancer [23, 33]. However, there are few studies on the degradation pathway of the BGN protein. Further exploration of BGN metabolism will provide more favorable evidence for the diagnosis and treatment of the PM of GC in the future. Existing research indicates that the ubiquitin-proteasome pathway is a very important factor in the regulation of tumor immunity, tumorigenesis, development, and prognosis. This is a vital pathway for protein degradation in eukaryotic cells, and approximately 80%-90% of proteins involved in cell function are degraded through the ubiquitin-proteasome pathway [20]. Our studies further indicated that the BGN protein could be ubiquitinated by FBXW7. These discoveries provide a theoretical basis for the prognostic evaluation, diagnosis, and treatment of PM in GC patients.

## 5. Conclusions

In summary, SEMA3B-AS1 inhibits the PM of GC by regulating the expression of the BGN protein. The specific mechanism is that SEMA3B-AS1 promotes the transcription and expression of FBXW7 by binding HMGB1 in the nucleus, and FBXW7 degrades the BGN protein via the ubiquitin-proteasome pathway. Therefore, SEMA3B-AS1 may be a novel and promising therapeutic target and biological marker for GC patients with PM.

## Figures and Tables

**Table 1:** The correlation of SEMA3B-AS1 expression with the clinicopathological parameters of GC patients (*n* = 50).

Parameters	*n* (%)	SEMA3B-AS1 expression		*p* value
		Low	High	
Gender				
Male	34 (68)	25	9	0.726
Female	16 (32)	11	5	
Age				
≥60 years	34 (68)	24	10	0.746
<60 years	16 (32)	12	4	
Cancer grade				
Moderate/well	10 (20)	6	4	0.345
Poor	40 (80)	30	10	
Cancer size (cm)				
≥5	13 (26.0)	13	0	0.009
<5	37 (74.0)	23	14	
T stage				
T1-2	11 (22.0)	4	7	0.003
T3-4	39 (78.0)	32	7	
Lymphatic metastasis				
N0-1	23 (46.0)	13	10	0.024
N2-3	27 (54.0)	23	4	
Distant metastasis				
Yes	11 (22.0)	11	0	0.019
No	39 (88.0)	25	14	
TNM stage				
I/II	17 (34.0)	7	10	<0.001
III/IV	33 (66.0)	29	4	
CEA (ng/mL)				
≥5	9 (18.0)	6	3	0.694
<5	41 (82.0)	30	11	
CA19-9 (u/mL)				
<37	40 (80.0)	28	12	0.529
≥37	10 (20.0)	8	2	
PM				
Yes	19 (38.0)	18	1	0.005
No	31 (62.0)	18	13	

Remark: tumor staging guidelines: AJCC Cancer Staging Manual (8th). Abbreviations: TNM: tumor-node-metastasis; CEA: carcinoembryonic antigen; CA19-9: carbohydrate antigen 19-9; T stage: tumor invasion stage; PM: peritoneal metastasis. *p* < 0.05: statistically significant.

**Table 2:** Univariate and multivariate analyses of clinicopathologic parameters correlated with progression-free survival (PFS) and overall survival (OS).

Parameters	Overall survival						Progression-free survival					
	Univariate analysis			Multivariate analysis			Univariate analysis			Multivariate analysis		
	HR	95% CI	*p* value	HR	95% CI	*p* value	HR	95% CI	*p* value	HR	95% CI	*p* value
Gender (male vs. female)	0.799	0.378-1.691	0.557				0.833	0.393-1.763	0.629			
Age (≥60 years vs. <60 years)	0.708	0.327-1.532	0.381				0.709	0.328-1.533	0.382			
Cancer grade (poor vs. moderate/well)	3.248	0.986-10.701	0.053				3.386	1.028-11.155	0.045	1.533	0.342-6.886	0.577
Cancer size (cm) (≥5 vs. <5)	1.781	0.834-3.801	0.136				1.752	0.821-3.738	0.147			
T stage (T3-4 vs. T1-2)	2.845	0.988-8.192	0.053				2.935	1.020-8.455	0.046	1.519	0.184-12.522	0.698
Lymphatic metastasis (N2-3 vs. N0-1)	4.083	1.810-9.208	0.001	5.204	1.641-16.507	0.005	4.029	1.785-9.902	0.001	4.791	1.509-15.209	0.008
TNM stage (III/IVVSI/II)	3.713	1.501-9.185	0.005	0.522	0.091-3.343	0.518	3.758	1.520-9.293	0.004	0.513	0.085-3.087	0.466
CEA (ng/mL) (≥5 vs. <5)	1.601	0.687-3.732	0.275				1.625	0.697-3.786	0.261			
CA19-9 (u/mL) (≥37 vs. <37)	2.927	1.319-6.495	0.008	2.191	0.635-7.553	0.214	2.769	1.244-6.164	0.013	1.531	0.431-5.435	0.51
SEMA3B-AS1 expression (high vs. low)	0.113	0.034-0.383	<0.001	0.113	0.021-0.603	0.011	0.112	0.033-0.376	<0.001	0.123	0.023-0.662	0.015
PM (no vs. yes)	0.245	0.111-0.539	<0.001	0.25	0.077-0.814	0.021	0.225	0.102-0.493	<0.001	0.193	0.061-0.615	0.005

Remark: tumor staging guidelines: AJCC Cancer Staging Manual (8th). Abbreviations: TNM: tumor-node-metastasis; CEA: carcinoembryonic antigen; CA19-9: carbohydrate antigen 19-9; T stage: tumor invasion stage; PM: peritoneal metastasis. *p* < 0.05: statistically significant.

## Data Availability

The data used to support the findings of this study are available from the corresponding author upon request.

## Abbreviations

GC: Gastric cancer
BGN: Biglycan
EMT: Epithelial-mesenchymal transition
SEMA3B-AS1: Long noncoding RNA SEMA3B-AS1
NC: Negative control
qRT-PCR: Quantitative reverse transcription polymerase chain reaction
PM: Peritoneal metastasis
PMT: Peritoneal metastasis tissue
LNM: Lymph node metastasis
TNM: Tumor-node-metastasis
DM: Distant metastasis
CA19-9: Carbohydrate antigen 19-9
CEA: Carcinoembryonic antigen
IHC: Immunohistochemistry
IP: Immunoprecipitation
co-IP: Coimmunoprecipitation
RIP: RNA-binding protein immunoprecipitation
ChIP: Chromatin immunoprecipitation
ChIRP: Chromatin isolation by RNA purification chromatin isolation by RNA purification
Lv-Oe-SEMA3B-AS1: Lentivirus-mediated overexpression of SEMA3B-AS1
Lv-ON-SEMA3B-AS1: Lentivirus-mediated negative control of SEMA3B-AS1
PO-SEMA3B-AS1: pCDNA overexpression of SEMA3B-AS1
PN-SEMA3B-AS1: pCDNA-negative control of SEMA3B-AS1
Lv-SH-SEMA3B-AS1: Lentivirus-mediated knockdown of SEMA3B-AS1
Lv-SHNC-SEMA3B-AS1: Lentivirus-mediated negative control of SEMA3B-AS1
FBXW7: F-box and WD repeat domain-containing 7
Lv-Oe-FBXW7: Lentivirus-mediated overexpression of FBXW7
Lv-ON-FBXW7: Lentivirus-mediated negative control of FBXW7
SH-FBXW7: pCDNA knockdown of FBXW7
SHNC-FBXW7: pCDNA-knockdown negative control of FBXW7
HMGB1: High mobility group box 1.

## Supplementary Materials

Supplementary 1 Supplementary Table 1: related gene primers mentioned in this article.

Supplementary 2 Supplementary Table 2: annotation information for RNA pull-down mass spectrometry.
