# Supplementary material for: The lncRNA SEMA3B-AS1/HMGB1/FBXW7 Axis Mediates the Peritoneal Metastasis of Gastric Cancer by Regulating BGN Protein Ubiquitination
Source: Oxid Med Cell Longev. 2022 Feb 27;2022:5055684. doi: 10.1155/2022/5055684 (PMC8902634; doi:10.1155/2022/5055684)
Supplement: Supplementary 1 — Supplementary Table 1: related gene primers mentioned in this article. [file 5055684.f1.docx]

**Supplementary table 1** Related gene primers mentioned in this article

| Gene | Sequence |
| --- | --- |
| lncRNASEMA3B-AS1 | F:5'-GCCTCAGTTGGGAATGACCT-3'. |
|  | R:5'-TCCCTCCAGAGAAGTACCCC-3′ |
| FBXW7 | F:5’-GTGGGACATACAGGTGGA-3′ |
|  | R:5’-CAACGCACAGTGGAAGTA-3′ |
| FBXW7-RNAi | ACCTACAGACTAAGAAGGAAA. |
| BGN | F:5’-AGGAGGCGGTCCATAAGAAT-3′ . |
|  | R:5’-AGGGTTGAAAGGCTGGAAAT-3′ |
| LncRNASEMA3B-AS1 FISH probe sequence: (5’-3’) | agactcagct+tcaggacaagacaag+tacccctcat  +gttacaaattatattcac+tcagg+tggacttc. |
| HMGB1 | F:5’-TTTCAAACAAAGATGCCACA-3’ |
|  | R:5’-GTTCCCTAAACTCCTAAGCAGATA-3' |
| GAPDH | F:5’-GGAAGCTTGTCATCAATGGAAATC-3’. |
|  | R:5’-TGATGACCCTTTTGGCTCCC-3’. |
| SEMA3B-AS1-CHIRP probe | |
| lnc40024683 | AGTGATTCCCTTTGCCTGCT-/3bio/ |
| lnc40024684 | TGTGGAGGGAGAGGTTGAGA-/3bio/ |
| lnc40024685 | TTTCGGCTCTGGGTTTGGTG-/3bio/ |
| lnc40024686 | ACGTTCACCAGACTCAGCTT-/3bio/ |
| lnc40024686-NC | TACGCACCACTAGTCGTACT-/3bio/ |
| SEMA3B-AS1-RNApulldown sequence | AACCCCAGCTCCAGGGGCTCAGCAGGCAAAGGGAATCACTGAGTGGGGGCACCACCCGTGGACTCCAATATCTCAACCTCTCCCTCCACAGGTTGGAGGTGGGAGGAACAACCCCCACCAAACCCAGAGCCGAAAACTGAGGGAGTTTTACAGACAGGACGGAGCTCCTGCACCTCGGAGCCTCAGTTGGGAATGACCTGGGGTCTTGTCCTGAAGCTGAGTCTGGTGAACGTGCCCCATTTGTAACATGAGGGGTACTTCTCTGGAGGGACTGTATGTTGACAGTGGCAGAGTGGAGCCCTGAAGTCCACCTGAGTGAATATACCAGGGCTTGAGAA |
| HMGB1-RNAi(52641-1) | GCAGATGACAAGCAGCCTTAT |
| HMGB1-RNAi(52642-1) | GCTGCAGCTTATACGAAATAA |
| HMGB1-RNAi(70894-1) | TCGGGAGGAGCATAAGAAGAA |
