# Supplementary material for: The lncRNA SEMA3B-AS1/HMGB1/FBXW7 Axis Mediates the Peritoneal Metastasis of Gastric Cancer by Regulating BGN Protein Ubiquitination
Source: Oxid Med Cell Longev. 2022 Feb 27;2022:5055684. doi: 10.1155/2022/5055684 (PMC8902634; doi:10.1155/2022/5055684)
Supplement: Supplementary 2 — Supplementary Table 2: annotation information for RNA pull-down mass spectrometry. [file 5055684.f2.docx]

Supplementary table 2 Annotation information for RNA pulldown mass spectrometry

| RNA/NC | pro t_acc | Gene symbol |
| --- | --- | --- |
| sp | P08779 | K1C16 |
| sp | Q04695 | K1C17 |
| sp | P27695 | APEX1 |
| sp | Q13509 | TBB3 |
| sp | P04792 | HSPB1 |
| sp | Q12906 | ILF3 |
| sp | Q06830 | PRDX1 |
| sp | O43684 | BUB3 |
| sp | P27816 | MAP4 |
| sp | O60763 | USO1 |
| sp | Q9H3N1 | TMX1 |
| sp | O75439 | MPPB |
| sp | Q9UHF7 | TRPS1 |
| sp | Q13409 | DC1I2 |
| sp | P18754 | RCC1 |
| sp | P31930 | QCR1 |
| sp | P61224 | RAP1B |
| sp | P23193 | TCEA1 |
| sp | P22314 | UBA1 |
| sp | P54727 | RD23B |
| sp | P21266 | GSTM3 |
| sp | P13489 | RINI |
| sp | Q13151 | ROA0 |
| sp | P22695 | QCR2 |
| sp | O75947 | ATP5H |
| sp | P09429 | HMGB1 |
| sp | P18085 | ARF4 |
| sp | P60866 | RS20 |
| sp | P16989 | YBOX3 |
| sp | Q8WVX9 | FACR1 |
| sp | P23919 | KTHY |
| sp | P24539 | AT5F1 |
| sp | Q15056 | IF4H |
| sp | Q9HB71 | CYBP |
| sp | P51149 | RAB7A |
| sp | Q9H6Z4 | RANB3 |
| sp | Q15286 | RAB35 |
| sp | O43242 | PSMD3 |
| sp | P60891 | PRPS1 |
| sp | P78347 | GTF2I |
| sp | P00390 | GSHR |
| sp | Q9H0U4 | RAB1B |
| sp | B5ME19 | EIFCL |
| sp | P0DME0 | SETLP |
| sp | P43686 | PRS6B |
| sp | P41250 | GARS |
| sp | Q14103 | HNRPD |
| sp | P11177 | ODPB |
| sp | O43837 | IDH3B |
| sp | O75396 | SC22B |
| sp | P22102 | PUR2 |
| sp | P62280 | RS11 |
| sp | P35250 | RFC2 |
| sp | P39748 | FEN1 |
| sp | P62263 | RS14 |
| sp | P22234 | PUR6 |
| sp | P11279 | LAMP1 |
| sp | P51991 | ROA3 |
| sp | Q9Y281 | COF2 |
| sp | P62191 | PRS4 |
| sp | Q00796 | DHSO |
| sp | P62942 | FKB1A |
| sp | Q9H9B4 | SFXN1 |
| sp | Q00839 | HNRPU |
| sp | P41252 | SYIC |
| sp | Q9BSJ8 | ESYT1 |
| sp | P62753 | RS6 |
| sp | Q13162 | PRDX4 |
| sp | P36957 | ODO2 |
| sp | P82979 | SARNP |
| sp | O60256 | KPRB |
| sp | O14979 | HNRDL |
| sp | P35613 | BASI |
| sp | Q13630 | FCL |
| sp | Q15785 | TOM34 |
| sp | Q96KG9 | SCYL1 |
| sp | P25789 | PSA4 |
| sp | P30043 | BLVRB |
| sp | P36873 | PP1G |
| sp | O15347 | HMGB3 |
| sp | Q8TCT9 | HM13 |
| sp | O75844 | FACE1 |
| sp | O95197 | RTN3 |
| sp | P26196 | DDX6 |
| sp | P51571 | SSRD |
| sp | Q9Y3A5 | SBDS |
| sp | Q8IYT4 | KATL2 |
| sp | Q9H9S3 | S61A2 |
| sp | Q8N138 | ORML3 |
| sp | P09661 | RU2A |
| sp | Q8NFQ8 | TOIP2 |
| sp | P78310 | CXAR |
| sp | P35637 | FUS |
| sp | P35237 | SPB6 |
| sp | O14818 | PSA7 |
| sp | P99999 | CYC |
| sp | P62273 | RS29 |
| sp | P08670 | VIME |
| sp | O43819 | SCO2 |
| sp | Q9NP72 | RAB18 |
| sp | O75223 | GGCT |
| sp | P05388 | RLA0 |
| sp | P62081 | RS7 |
| sp | P27105 | STOM |
| sp | Q9H7Z7 | PGES2 |
| sp | Q9NQC3 | RTN4 |
| sp | O15260 | SURF4 |
| sp | Q9Y2Z0 | SGT1 |
| sp | Q14108 | SCRB2 |
| sp | Q6YN16 | HSDL2 |
| sp | P12081 | HARS1 |
| sp | P16152 | CBR1 |
| sp | Q9UBT2 | SAE2 |
| sp | P46782 | RS5 |
| sp | Q5JWF2 | GNAS1 |
| sp | Q13057 | COASY |
| sp | Q15459 | SF3A1 |
| sp | P53597 | SUCA |
| sp | P62937 | PPIA |
| sp | Q9H2H9 | S38A1 |
| sp | Q8N163 | CCAR2 |
| sp | P51665 | PSMD7 |
| sp | Q13155 | AIMP2 |
| sp | Q13616 | CUL1 |
| sp | P29373 | RABP2 |
| sp | Q9Y4C1 | KDM3A |
| sp | P38606 | VATA |
| sp | Q92688 | AN32B |
| sp | Q9BT78 | CSN4 |
| sp | Q06210 | GFPT1 |
| sp | Q8WVY7 | UBCP1 |
| sp | P61221 | ABCE1 |
| sp | Q15005 | SPCS2 |
| sp | Q9NRN7 | ADPPT |
| sp | Q99715 | COCA1 |
| sp | P51153 | RAB13 |
| sp | P29508 | SPB3 |
| sp | A2RTX5 | SYTC2 |
| sp | P29590 | PML |
| sp | P18206 | VINC |
| sp | Q16659 | MK06 |
| sp | P46940 | IQGA1 |
| sp | Q16822 | PCKGM |
| sp | P05109 | S10A8 |
| sp | P10644 | KAP0 |
| sp | P37108 | SRP14 |
| sp | O00232 | PSD12 |
| sp | Q9ULH7 | MRTFB |
| sp | P09382 | LEG1 |
| sp | Q9GZT8 | NIF3L |
| sp | P0C221 | CC175 |
| sp | Q99497 | PARK7 |
| sp | P42126 | ECI1 |
| sp | O00303 | EIF3F |
| sp | O60568 | PLOD3 |
| sp | P30044 | PRDX5 |
| sp | Q9Y697 | NFS1 |
| sp | P02545 | LMNA |
| sp | Q8N7U6 | EFHB |
| sp | Q01581 | HMCS1 |
| sp | P17812 | PYRG1 |
| sp | Q12770 | SCAP |
| sp | P67809 | YBOX1 |
| sp | Q12769 | NU160 |
| sp | Q15645 | PCH2 |
| sp | P12273 | PIP |
| sp | P36507 | MP2K2 |
| sp | Q99436 | PSB7 |
| sp | P60510 | PP4C |
| sp | Q9NUJ1 | ABHDA |
| sp | Q15051 | IQCB1 |
| sp | P11413 | G6PD |
| sp | Q8N6H7 | ARFG2 |
| sp | Q08257 | QOR |
| sp | P40938 | RFC3 |
| sp | P25685 | DNJB1 |
| sp | Q96PK6 | RBM14 |
| sp | P62333 | PRS10 |
| sp | Q6NUK1 | SCMC1 |
| sp | Q9BRP8 | PYM1 |
| sp | P46060 | RAGP1 |
| sp | Q9UKA9 | PTBP2 |
| sp | P52292 | IMA1 |
| sp | P52907 | CAZA1 |
| sp | Q07866 | KLC1 |
| sp | P61289 | PSME3 |
| sp | P51858 | HDGF |
| sp | Q99460 | PSMD1 |
| sp | O00442 | RTCA |
| sp | P60953 | CDC42 |
| sp | Q96G03 | PGM2 |
| sp | A0A0C5B5G6 | MOTSC |
| sp | P52943 | CRIP2 |
| sp | Q9Y5X3 | SNX5 |
| sp | Q9P2W9 | STX18 |
| sp | P00568 | KAD1 |
| sp | P12814 | ACTN1 |
| sp | P06132 | DCUP |
| sp | P14678 | RSMB |
| sp | Q9C0E8 | LNP |
| sp | P10515 | ODP2 |
| sp | P35249 | RFC4 |
| sp | A3KN83 | SBNO1 |
| sp | Q8TAG9 | EXOC6 |
| sp | Q14011 | CIRBP |
| sp | Q9Y4R7 | TTLL3 |
| sp | O15440 | MRP5 |
| sp | Q5JU69 | TOR2A |
| sp | A0A0U1RQE8 | GLYLB |
